# Posterior Reversible Encephalopathy Syndrome and Reversible Cerebral Vasoconstriction Syndrome Associated With Relugolix Therapy for Giant Uterine Fibroids and Anemia: A Case Report

**DOI:** 10.7759/cureus.106157

**Published:** 2026-03-30

**Authors:** Moe Kawashima, Kenji Yoshida, Ryuji Suzuki, Norihito Kamo, Jun Miyashita

**Affiliations:** 1 Clinical Training Center, Shirakawa Kosei General Hospital, Fukushima, JPN; 2 Department of General Medicine, Shirakawa Satellite for Teaching and Research (STAR), Fukushima Medical University, Fukushima, JPN; 3 Department of Gynecology, Shirakawa Kosei General Hospital, Fukushima, JPN

**Keywords:** iron deficiency anemia (ida), posterior reversible encephalopathy syndrome (pres), relugolix, reversible cerebral vasoconstriction syndrome (rcvs), uterine fibroid

## Abstract

Posterior reversible encephalopathy syndrome (PRES) is a clinico-radiological condition characterized by reversible vasogenic edema. Reversible cerebral vasoconstriction syndrome (RCVS) is a disorder characterized by segmental narrowing of the cerebral arteries that causes severe headaches. We report a case of PRES and RCVS development in a 52-year-old woman treated with a gonadotropin-releasing hormone antagonist, relugolix, and oral iron supplementation for uterine fibroids and iron deficiency anemia. In this case, relugolix may have contributed to the onset of PRES and RCVS through estrogen suppression and the resulting endothelial dysfunction. Additionally, gradual treatment of anemia over two months with oral iron alone may have played a role in this case, given that increased intravascular volume and viscosity can precipitate vascular injury and vasoconstriction. To our knowledge, this represents the first reported case of concurrent PRES and RCVS associated with relugolix therapy.

## Introduction

Posterior reversible encephalopathy syndrome (PRES) is a clinico-radiological entity characterized by reversible vasogenic edema that typically affects the posterior white matter. Known triggers include acute hypertension, gynecological conditions, blood transfusions, organ transplantation, invasive procedures, and various medications [1-3].

Reversible cerebral vasoconstriction syndrome (RCVS) is a cerebrovascular disorder characterized by segmental constriction of the cerebral arteries that causes sudden and severe headaches and may lead to ischemic or hemorrhagic complications. Although PRES and RCVS are distinct entities, they often coexist and share overlapping pathophysiological mechanisms, such as endothelial dysfunction and impaired cerebrovascular autoregulation [4].

Relugolix is a gonadotropin-releasing hormone (GnRH) receptor antagonist that directly blocks pituitary GnRH receptors, resulting in rapid suppression of luteinizing hormone (LH) and follicle-stimulating hormone (FSH) without an initial hormone surge. In contrast, leuprolide and other GnRH agonists initially stimulate the receptor, producing a transient hormonal flare before suppression. Although GnRH agonists are potential triggers, no cases of PRES and RCVS caused by antagonists have been reported [5]. Oral contraceptives have no effect on uterine fibroid growth; however, GnRH antagonists are effective for shrinking fibroids. Therefore, because of their convenience and superior efficacy, GnRH antagonists have become the first-line treatment for uterine fibroids [6]. However, the increased use of GnRH antagonists may be associated with an increase in the occurrence of complications [7]. We present a case of concomitant PRES and RCVS that developed in a patient who used relugolix, a GnRH antagonist, and oral iron supplementation for large uterine fibroids and anemia.

## Case presentation

**Figure 1:**
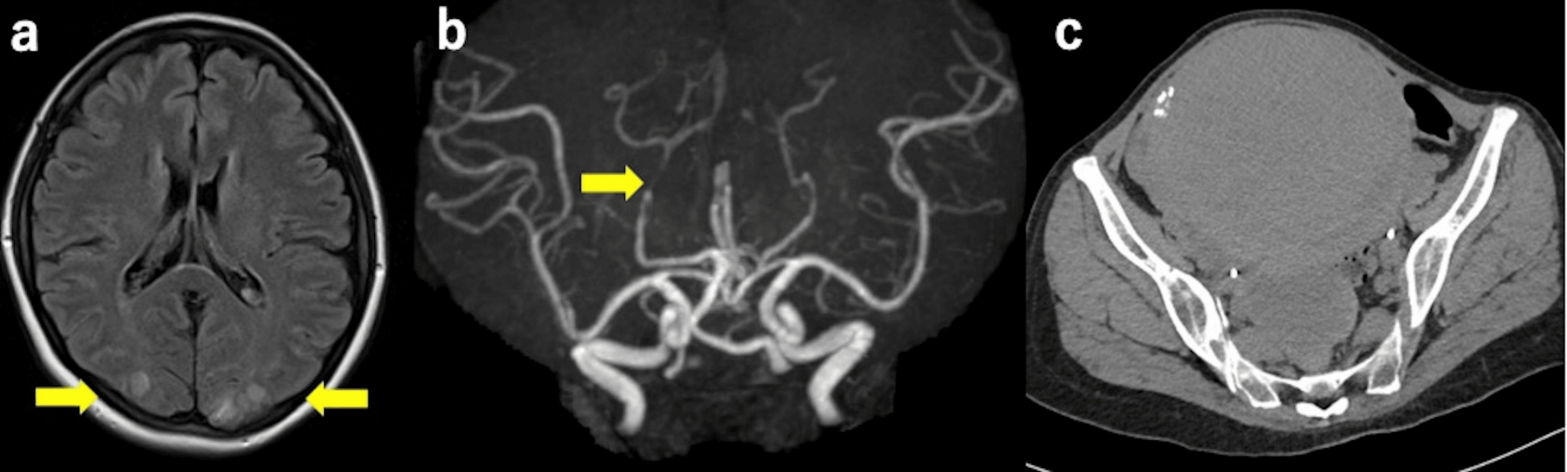
Axial FLAIR MRI (1.5 T; repetition time, 10,000 ms; echo time, 102 ms) on the day of PRES onset (a) shows bilateral hyperintensity in the occipital subcortical white matter (arrows) (a). MRA on the same day (b) demonstrates segmental narrowing of the posterior cerebral arteries consistent with vasospasm (arrow). Abdominal CT on the same day as PRES onset (c) demonstrates large uterine fibroids. FLAIR, fluid-attenuation inversion recovery; MRI, magnetic resonance imaging; PRES, posterior reversible encephalopathy syndrome; MRA, Magnetic resonance angiography; CT, computed tomography.

A 52-year-old woman presented to our gynecology department with menorrhagia and prolonged menstruation in September 2024. She had a known history of uterine fibroids (Figure 1c) and was not using any regular medications. She had smoked approximately 15 cigarettes daily for 25 years. Blood pressure at the initial visit was 128/68 mmHg. Initial laboratory tests revealed severe microcytic anemia (hemoglobin (Hb), 4.1 g/dL; mean corpuscular volume, 55.4 fL). Abdominal computed tomography (CT) showed uterine fibroids with a diameter of 14 cm. Consequently, oral relugolix (40 mg/day) and ferrous citrate (500 mg/day) were initiated. Relugolix was administered for 74 days until the onset of the event, and no estrogen add-back therapy was provided. Hb levels improved to 7.9 g/dL 53 days after the initiation of iron supplementation and further increased to 11.8 g/dL after 85 days of treatment. An abdominal hysterectomy as definitive treatment was planned.

In November 2024, prophylactic bilateral ureteral stents were placed preoperatively by physicians in the urology department. That same evening, while bathing at home, a thunderclap headache, vomiting, and visual disturbances developed, followed by impaired consciousness and generalized tonic-clonic seizures during ambulance transport to the hospital.

On arrival, her blood pressure was 172/97 mmHg; heart rate, 77 bpm; temperature, 36.0°C, and oxygen saturation, 98% in room air. She was alert (Glasgow Coma Scale score, 15) and seizure-free at presentation. A neurological examination indicated no focal deficits.

Laboratory tests revealed the following results: white blood cell count, 5,800/μL; Hb, 11.8 g/dL; platelets, 95,000/μL; creatinine, 0.74 mg/dL; sodium, 141 mmol/L; potassium, 3.5 mmol/L; and glucose, 126 mg/dL. The cerebrospinal fluid examination indicated the following: white blood cell count, 4/μL; protein, 32 mg/dL; and glucose, 58 mg/dL (Table 1). Thrombocytopenia was also observed. Additional blood tests and bone marrow aspiration were performed, leading to a diagnosis of immune thrombocytopenia (ITP). Hypokalemia resolved spontaneously and returned to normal levels the following day with observation alone. The mildly elevated glucose level at admission (126 mg/dL) was considered a transient stress response. Fluid-attenuated inversion recovery magnetic resonance imaging of the brain showed hyperintensities in the subcortical white matter of bilateral occipital lobes (Figure 1a). Magnetic resonance angiography revealed segmental narrowing of the right posterior cerebral artery (Figure 1b). These findings were consistent with those of PRES and concurrent RCVS.

**Table 1:** Initial laboratory findings demonstrating hypokalemia and low levels of serum iron. Renal and liver function tests were within normal limits.

Section	Test name	Result	Reference range
Complete blood count (CBC)	White blood cells (×10^6^/µL)	5.8	3.3-8.6
	Red blood cells (×10^6^/µL)	5.27	3.63-4.92
	Hemoglobin (g/dL)	11.8	11.6-14.8
	Hematocrit (%)	40.2	35.1-44.4
	Platelets (×10^3^/µL)	95	158-348
Complete metabolic panel	Lactate dehydrogenase (U/L)	209	124-224
	Sodium (mmol/L)	141	138-145
	Potassium (mmol/L)	3.5	3.6-4.8
	Chloride (mmol/L)	102	101-108
	Blood urea nitrogen (mg/dL)	10.3	8.0-20.0
	Creatinine ratio (mg/dL)	0.74	0.30-1.10
	Aspartate aminotransferase (U/L)	23	13-30
	Alanine aminotransferase (U/L)	15	7-23
	Alkaline phosphatase (U/L)	81	38-113
	C-reactive protein (mg/dL)	0.04	0.000-0.140
	Glucose (mg/dL)	126	73-109
	Serum iron (µg/dL)	31	40-188
	Ferritin (ng/mL)	31	4-100
	Total iron binding capacity (µg/dL)	350	270-470
Cerebrospinal fluid examination	Color	Colorless	
	Cells (/µL)	4	
	Protein (mg/dL)	32	10-50
	Glucose (mg/dL)	58	50-80

The patient was treated with intravenous nicardipine, which was later transitioned to oral amlodipine. Levetiracetam was initiated as seizure prophylaxis. Relugolix and iron supplementation were discontinued, bathing was restricted, and surgery was postponed.

**Figure 2:**
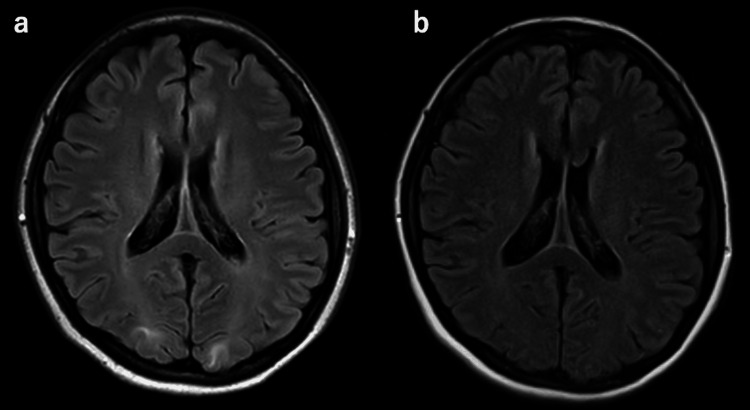
Axial FLAIR MRI (1.5 T; repetition time, 11,000 ms; echo time, 140 ms) on day 3 (a) and day 17 (b) (1.5 T; repetition time, 10,000 ms; echo time, 102 ms). (a) Partial resolution of lesions after 3 days. (b) Complete resolution of lesions by day 17. FLAIR, fluid-attenuation inversion recovery; MRI, magnetic resonance imaging.

**Figure 3:**
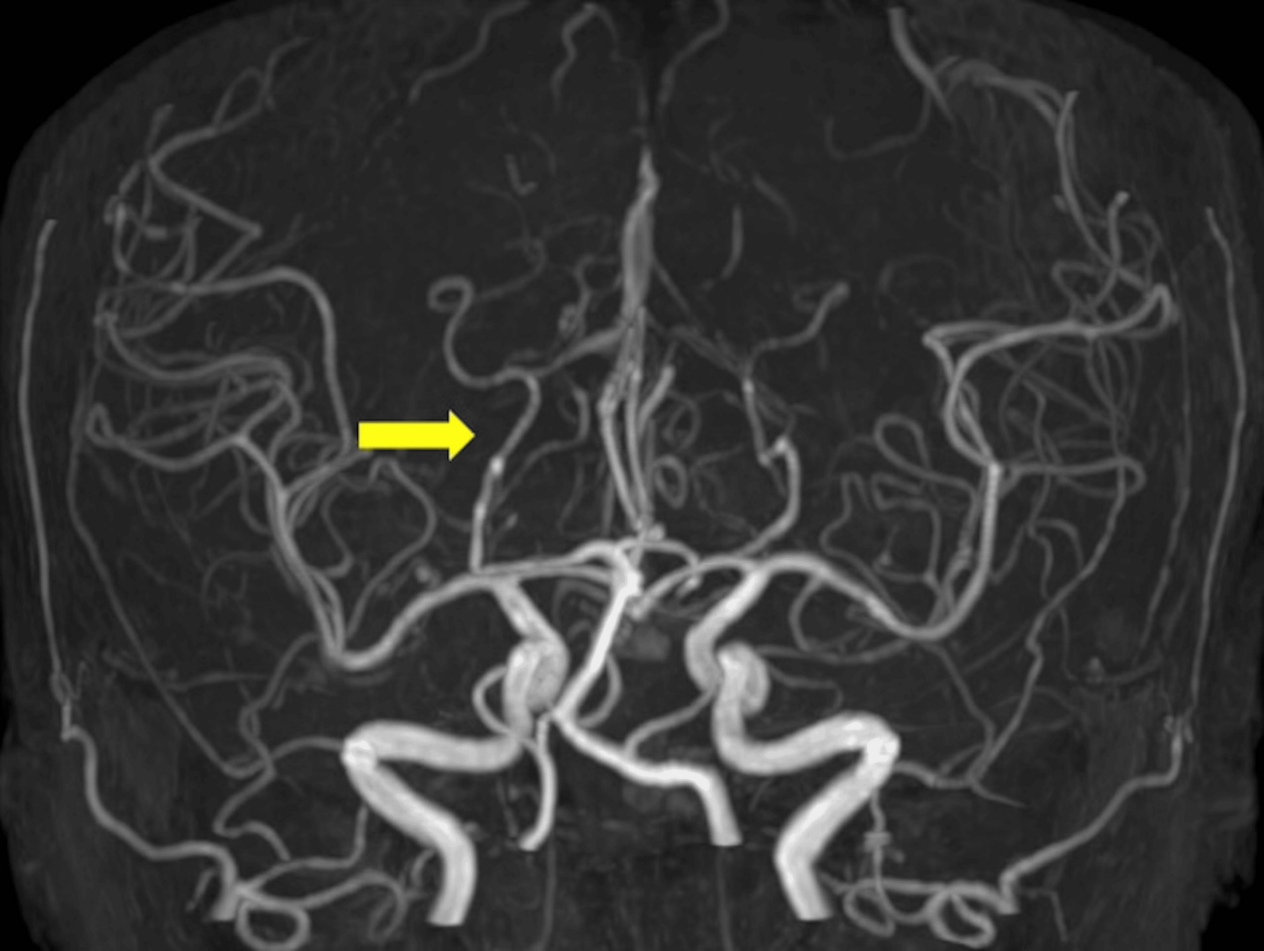
MRA (1.5 T; repetition time, 11,000 ms; echo time, 140 ms) on day 3 demonstrates improvement in stenosis of the posterior cerebral artery (arrow). MRA, magnetic resonance angiography.

Her symptoms resolved rapidly, and follow-up magnetic resonance imaging on day 3 showed marked improvement; complete resolution occurred by day 17 (Figure 2). Magnetic resonance angiography also showed improvement in posterior cerebral artery stenosis on day 3 (Figure 3). The patient was discharged on day 20. On day 104, the patient underwent abdominal hysterectomy, which was initiated via open surgery and allowed the removal of a 3 kg uterus. A histological examination revealed spindle-shaped smooth muscle cells without atypia, thus confirming the diagnosis of uterine fibroids. In December 2025, the patient was symptom-free, and levetiracetam had been discontinued.

## Discussion

PRES is a disease characterized by vasogenic edema, predominantly in the posterior white matter, that is often reversible. However, it may also be associated with intracerebral hemorrhage, cerebral infarction, or subarachnoid hemorrhage; therefore, prompt management during the acute phase and recurrence prevention are necessary [8]. RCVS is a disorder in which transient constriction of the cerebral arteries causes sudden and severe thunderclap headaches. Additionally, RCVS is reversible, but it can be complicated by cerebral hemorrhage and other conditions. Both PRES and RCVS are disorders involving abnormalities of the cerebral vasculature, and they are considered overlapping syndromes. Their management focuses on eliminating background precipitating factors, such as hypertension and medications, as well as disease-specific treatments, including antihypertensive therapy, anticonvulsants, and vasodilators [4]. This case highlights two potential factors that may contribute to PRES and RCVS: GnRH antagonist therapy and anemia treatment. Relugolix is an oral nonpeptide GnRH antagonist that was approved in Japan in 2019 for the treatment of uterine fibroids and is generally well tolerated. GnRH antagonists are used to shrink uterine fibroids and treat endometriosis; additionally, they have been proven to significantly reduce hypermenorrhea [6]. Due to their convenience and excellent efficacy, GnRH antagonists have become the first-line treatment for uterine fibroids in patients approaching menopause. Additionally, when surgical treatment for uterine fibroids is necessary, GnRH antagonist therapy may be administered for three to four months before surgery [9]. This approach aims to reduce the size of fibroids, improve preoperative anemia, decrease intraoperative blood loss, and lower the rate of surgical complications. Therefore, opportunities for GnRH antagonist use are increasing annually. Relugolix, a combination formulation of relugolix and estradiol, is available in the United States, Canada, and Europe. Hypertension is the most common precipitating factor for PRES [10], followed by gynecological disorders such as eclampsia and hemolysis, elevated liver enzymes, and low platelets syndrome, as well as procedures such as transplantation and transfusion [11]. Although PRES has been reported with the use of GnRH agonists, such as leuprolide [5], only one case related to a GnRH antagonist has been reported [12]. In that case, PRES developed on day 8 after the initiation of a GnRH antagonist for a patient who had been using relugolix and iron supplementation [12]. Although RCVS associated with GnRH agonists has also been reported [13], no cases related to GnRH antagonists have been described. GnRH agonists initially cause a transient increase in gonadotropin production attributable to pituitary stimulation. This increase is followed by desensitization, which reduces gonadotropin secretion from the pituitary gland and ultimately decreases estrogen levels. In contrast, GnRH antagonists reduce estrogen secretion by directly blocking GnRH receptors, thereby suppressing pituitary hormone release and decreasing gonadotropin production. Thus, both agents improve symptoms of uterine fibroids by decreasing estrogen levels. Estrogen suppression may promote vasoconstriction via decreased nitric oxide and prostacyclin production and increased endothelin release, potentially predisposing patients to PRES and RCVS.

Cases of PRES and RCVS related to anemia treatment, particularly after blood transfusion, have been reported [1,14]. Recently, cases of PRES and RCVS have been linked to gradual oral iron treatment [15-18]. Proposed mechanisms include increased intravascular volume and blood viscosity, which lead to endothelial injury and vasoconstriction [1,19].

In this case, the onset occurred after a urological procedure and after bathing. Previous studies have reported the development of PRES and RCVS following invasive procedures, suggesting that such procedures may act as potential triggers [1-3]. Similarly, multiple reports have described RCVS occurring in association with bathing [20]. Therefore, in patients at increased risk, such as those receiving relugolix or undergoing treatment for anemia, the potential risk associated with invasive procedures and bathing should be carefully considered.

In this case, similar to other reported cases of PRES and RCVS, management consisted of removal of the precipitating factors and symptomatic therapy. With this approach, the patient’s symptoms improved without complications. If PRES and RCVS occur during relugolix treatment, then discontinuation is likely necessary. Additionally, anemia treatment should be administered gradually and with caution in high-risk cases.

## Conclusions

This case highlights two important points. First, as a GnRH antagonist, relugolix may induce PRES and RCVS. Second, anemia treatment may be a risk factor for PRES and RCVS. In our patient, the combined effects of relugolix-induced endothelial dysfunction, anemia treatment, bathing, and preoperative procedures may have acted synergistically. Complications and adverse events may increase with increasing relugolix use. Therefore, clinicians should be aware of the potential risk of PRES and RCVS, particularly among patients receiving treatment with relugolix and iron supplements, and those who have undergone perioperative procedures.
